# Application of Near Infrared Spectroscopy to Enhance Safety and Individualize Distraction of Severely Contracted Joints in Far-Advanced Dupuytren’s Disease

**DOI:** 10.3390/jcm13144025

**Published:** 2024-07-10

**Authors:** Wibke Müller-Seubert, Aijia Cai, Raymund E. Horch

**Affiliations:** Department of Plastic and Hand Surgery and Laboratory for Tissue Engineering and Regenerative Medicine, University Hospital Erlangen, Friedrich-Alexander University Erlangen-Nürnberg (FAU), 91054 Erlangen, Germany

**Keywords:** imaging, near infrared spectroscopy, Dupuytren’s disease, skeletal distraction

## Abstract

**Background**: Slow distraction of contracted joints is a well-established treatment in far-advanced stages of Dupuytren’s disease (DD). To assess finger perfusion and avoid malperfusion, we studied near infrared spectroscopy (NIRS) to evaluate the maximum extent of distraction that would not harm microcirculation to the finger. This technique also allows an optimized treatment in accordance with sufficient blood perfusion during distraction. **Methods**: Eligible patients with stage IV finger contractures who needed treatment for Dupuytren’s contracture were included and prospectively investigated. The operation was performed with local anaesthesia. First, the Dupuytren strand of the treated finger was dissected in the palm to allow distraction. Under X-ray control, the distraction device was applied. Then, slow distraction of the treated joint was performed to evaluate the finger perfusion. To assess perfusion of the treated finger, NIRS was used to measure tissue oxygen saturation. If impaired finger perfusion was detected, traction was reduced until sufficient oxygen levels and perfusion patterns were reestablished. **Results**: NIRS was performed after application of the distraction device in seven cases. We treated six male and one female patient (mean age 70 years, range 51–80 years). Rapid distraction resulted in malperfusion of the treated fingers. Using NIRS proved to render reliable and reproducible information on finger perfusion and oxygenation in all seven patients. **Conclusions**: Application of NIRS enhances safety in the treatment of far-advanced DD finger contractures with an external skeletal distraction device. It is non-invasive, reproducible, easy to use and allows for an individualized adapted distraction velocity.

## 1. Introduction

Dupuytren’s disease (DD) is a benign fibromatosis of the palmar fascia that causes pathologic bands, which over time contract and cause flexion contractures of the small finger joints [1]. DD is a multifactorial disease—genetic predisposition, ethnic characteristics, alcohol intake, smoking, diabetes, repetitive trauma and exposure to vibration have been identified as risk factors for the development of DD [2]. Furthermore, DD has an estimated prevalence of 21% by the age of 65 years among Western populations [2]. The initial stages of DD, such as contour changes, wrinkles and dimples, are often ignored by the patients. Patients often search for medical help when later stages occur with painful nodes or increased flexion deformity of the fingers [1]. One reason might be an increasing flexion deformity of the ring and little finger metacarpophalangeal joint (MCPJ) of more than 50°, which results in clinically important hand disability [3].

Management of DD can range from minimal cord dissection to invasive surgical excision depending on the disease severity and the patient’s preference. Different treatment algorithms have been described, with an initial treatment of needle aponeurotomy, collagenase injection and fasciotomy, followed by fasciectomy depending on recurrence or limited effectiveness [4]. Although treatment with collagenase injections appears to be promising in some cases, there is a high recurrence rate associated with low patient satisfaction, especially when treating a contracture at the level of the PIP joint (PIPJ) [5]. This recurrence rate is partly due to the fact that although the collagenase injection enables lysis of the thickened cord, it does not eliminate the pathological cord completely, which is why pathological collagen remains in situ [5]. Thus, limited fasciectomy is the standard treatment in many cases [6]. Surgical treatment with limited fasciectomy has main complications such as paresthesia/numbness, nerve injury or neuropraxia or scar sequelae, but nevertheless, these complications are in general low (<1%) [7]. However, the frequency of complications depends on the severity of the disease, the affected joint type (MCPJ or PIPJ) and if multiple digits are involved [7]. Thus, other studies have reported higher complication rates with severe complications such as infections, delayed wound healing, and neurovascular and tendon injuries at 7% [8].

In the advanced stages, especially in recurrent disease, shortening arthrodesis represents an alternative approach [9]. Arthrodesis may be indicated in patients with a recurrence of the disease after previous operative treatments and who have a very high risk of recurrence or a poor outcome after another operative treatment. These patients are sometimes offered joint arthrodesis or amputation [10]. However, an arthrodesis compromises finger function [11]. In an earlier retrospective study, elective amputation was indicated in 2% of patients with DD [12]. Furthermore, a study reported more than 40 DD-associated amputations per year in France [13]. To avoid negative results such as phantom pain or painful neuroma, alternative methods for treatment of advanced stages in DD are necessary [14]. Given the propensity of relapsing contractures, pretreatment seems worthwhile. As the rapid extension of contracted joints can result in tension on vessels and nerves leading to ischemic disorders of the fingers or, in worst cases, to amputation, a staged approach is recommended [15]. Remarkably, a study showed an extremely high unplanned rate of finger amputations of 8% in patients who received first re-operation using limited fasciectomy after primary dermofasciectomy [16]. One reason might be a surgical disruption to the digital arteries [17]. Furthermore, extending a contracted joint too quickly can also injure the shortened vessels.

In the advanced stages, slow distraction using distraction devices has been shown to be effective [18,19,20,21,22]. Nevertheless, to enhance patient safety, the additional use of modern imaging tools is helpful to assess the perfusion after the application of the distraction device. Non-invasive tools have been shown to be effective in daily use in plastic surgery, for example, in postoperative flap monitoring [23,24].

Near infrared spectroscopy (NIRS) is a non-invasive tool that evaluates tissue oxygen saturation. As oxygenated and deoxygenated hemoglobin have separated spectral signatures in the visible and near-infrared light spectrum, NIRS measures the different absorption of red and near-infrared wavelengths. In this manner, NIRS can determine the proportion of oxygenated and deoxygenated hemoglobin in the examined tissue [25]. Briefly, near infrared light is transmitted onto the skin surface and reflected off the blood within the tissue. Due to the wavelength dependent difference of the oxygenated and deoxygenated light absorption of hemoglobin, the ratio from oxygenated to deoxygenated blood, and therefore the viability, can be determined by this method. Poorly perfused skin has a lower percentage of oxygenated hemoglobin than well-perfused skin [26,27]. The used NIRS device is handheld, has no direct patient contact, is easy to use and mobile. These features allow a versatile use of the device.

In this study, we present the use of NIRS in the context of joint distraction in far-advanced stages of DD.

## 2. Materials and Methods

**Figure 1:**
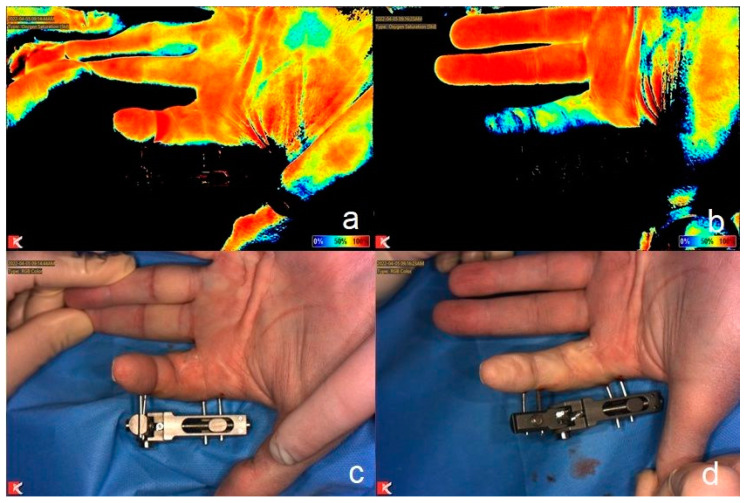
Measurement of tissue oxygen saturation using NIRS before (**a**) and after distraction (**b**) of the PIP joint of a left index finger with color scale ranging from dark blue (0% oxygenation) to red (100% oxygenation). Clinical picture before (**c**) and after distraction (**d**). Malperfusion of the finger is seen both in NIRS (**b**) and clinically (**d**).

The study was approved by the institutional ethics committee (Ethics Committee, FAU, 310_19 B9). Patients with advanced stages of M. Dupuytren (stadium III or IV) with a flexion deformity of the proximal interphalangeal joint (PIPJ) were included in the study by a consultant of the hand surgery department. The operation and the measurements were performed in the same two operation theaters under local anesthesia and has been described previously [28]. First, the Dupuytren strand of the treated finger was dissected in the palm to allow distraction. Under X-ray control, the distraction device was applied. Therefore, two self-drilling pins were applied into each the proximal and the middle phalanx of the treated finger to allow PIPJ mobilization. Then, the distraction device was applied, and slow distraction of the treated joint was performed to evaluate finger perfusion. To assess perfusion of the treated finger, near infrared imaging was used to measure tissue oxygen saturation. Near infrared reflectance-based imaging was performed at a standardized distance using Snapshot NIR^®^ (KENT Imaging Inc., Calgary, AB, Canada) to measure oxygen saturation of the treated finger. If malperfusion was detected, less traction was applied (Figure 1). The decision was obtained by the consultant who performed the operation. At the end of the operation, skin closure was performed using non-absorbable sutures. The study was performed according to the guidelines of Helsinki. Descriptive analysis was performed using Excel 2016 (Microsoft, Redmond, WA, USA).

## 3. Results

Seven cases are presented where NIRS was performed before, during and after application of the distraction device. We treated six male and one female western European patients (mean age 70 years, range 51–80 years) between 2019–2023. The distraction device was applied to six proximal interphalangeal joints (PIPJ) of the left little finger and one PIPJ of the left index finger.

Two fingers were classified as M. Dupuytren stadium III and five as stadium IV. Two patients suffered from recurrent disease. The duration of the illness was a minimum of two years.

**Figure 2:**
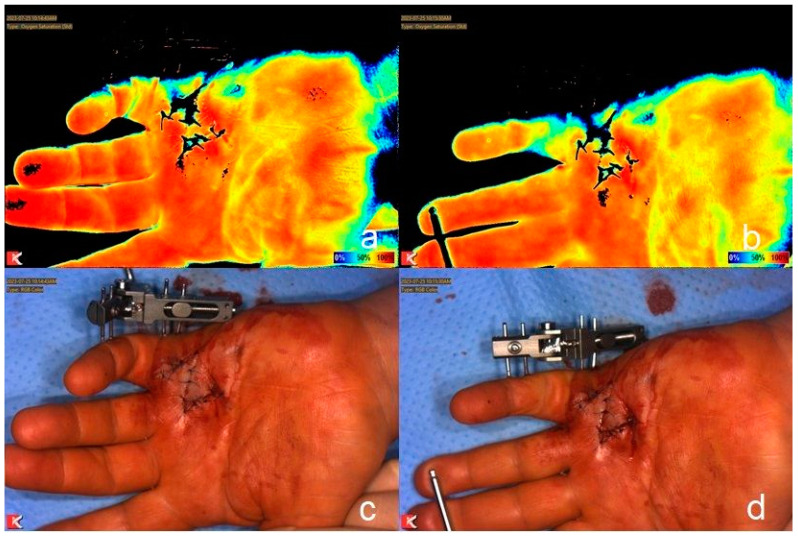
Measurement of tissue oxygen saturation using NIRS before (**a**) and after distraction (**b**) of the PIP joint of a left little finger with color scale ranging from dark blue (0% oxygenation) to red (100% oxygenation). Clinical picture before (**c**) and after distraction (**d**). Malperfusion of the finger is seen in NIRS (**b**) and less obvious clinically (**d**).

Malperfusion was seen after distraction of the treated joints, so the traction was reduced (Figure 2). After removal of the distraction device when full extension was achieved, no malperfusion or numbness of the treated was finger was detected.

Due to the staged approach, the patients received partial fasciectomy after removal of the device, so no further follow-up regarding the distraction procedure alone was possible.

## 4. Discussion

Treatment of far-advanced stages of DD remains a challenge for hand surgeons. Complications in surgical treatment of DD are relatively common and occur more often in recurrent disease compared to primary disease, especially in cases of nerve and artery injuries [29]. Higher complication rates in more severe stages of DD, especially circulation disorders or numbness, might be explained due to the more complex surgery [30]. Another explanation might be too rapid an extension of the shortened nerves and vessels.

Furthermore, complication rates increase in relation to the severity of the disease, especially when the PIPJ contracture is more than 60° [31]. Dias et al. reported similar results with a correlation of number of complications and the severity of the disease. In particular, numbness occurred in 46% of patients with contracted PIPJ and MCPJ compared to 36% in all patients [30]. Circulation disorders in severely affected PIPJ and MCPJ was seen in 17% of patients compared to 12% in all patients [30]. In many cases, postoperative neurapraxia or vascular compromise has been described [32]. In particular, the number of patients with neurapraxia has been reported to be at 9% [33]. Even though finger amputation is rare, a cumulative incidence of finger amputations has been described at 0.5% 30 days postoperatively and 0.6% 90 days postoperatively. In recurrent disease, this percentage is higher, with rates up to 8% [16].

In far-advanced stages, the application of a distraction device has been shown to be useful. Zolotov described a case series of four male patients treated with a distraction device without any ischemic disorders [15]. Kawakatsu presented a case report with a patient who was simultaneously treated with regional fasciectomy, skin grafting and distraction arthrolysis of the PIPJ [34]. Rajesh et al. described a staged approach using a distraction device; they reported no amputations [35]. Other distraction devices such as the “Pipster” has been described for successful treatment in a staged approach [36]. Messina et al. postulated that application of a continuous extension technique has eliminated finger amputations in severe Dupuytren’s contracture [18]. Due to these numerous studies that have successfully described skeletal distraction in DD, this study will not discuss this surgical technique per se.

Expansion of joint contractures in DD with a skeletal distraction device is also an established procedure in our clinic, which is mainly used in very advanced stages [20,22,28]. Nevertheless, regarding the development of new imaging tools in reconstructive surgery [24,37,38], the question is how this established procedure can be refined. Therefore, this study assesses if NIRS improves this well-established treatment.

To avoid malperfusion of the treated finger, joint contractures should be released slowly. In general, perfusion of tissue can be assessed clinically [39]. Nevertheless, early changes in perfusion can be subtle, so clinical observation might be difficult. It has been shown that NIRS was able to detect vascular compromise in flap surgery earlier than other monitoring methods, such as a physical examination or Doppler monitoring, and achieved a higher salvage rate in flap revision [40]. Furthermore, it detects a 30% decrease in tissue oxygen saturation before clinical changes are observable [41]. In the context of DD, NIRS has demonstrated improved tissue perfusion in terms of quantity and quality following early postoperative ergotherapy. After ergotherapy, all patients showed a relative increase in oxygen saturation of up to 20% [42].

The application of NIRS after application of a skeletal distraction device in far-advanced DD offers the possibility to improve the outcome when poor perfusion is detected. The distraction device can be set to its original position so that distraction is reduced on the treated finger to normalize perfusion. NIRS is easy to use, non-invasive and does not require specific clinical training. As it does not require intravenous injections, there is no additional cost for disposables. Furthermore, the used device is wireless and is easy to transport. It has a short measurement time that is acceptable for the patient and that does not prolong the operation time significantly. Furthermore, it has been shown to have high sensitivity and specificity in flap monitoring [24].

Perfusion assessment using Indocyanine angiography remains the gold standard in flap surgery and helps to reduce fat necrosis and partial flap loss [37,38]. However, in contrast to NIRS, it requires intravenous application of a dye, the device is less portable and the assessment cannot be repeated within a short time as the dye must be washed out.

The portable NIRS device we used has some limitations. Patients with darkly pigmented skin might be more difficult to examine. The higher concentration of melanin in the skin absorbs more wavelengths of light, and less light waves are reflected back to the device; this might be a reason that measurements of hemoglobin quantities is less accurate [43]. Furthermore, the measurements are sensitive to highly contoured surfaces, light glare and foreign materials. Sutures and hair absorb wavelengths in the NIR spectrum result in the dampening of the reflected wavelengths that are used for the interpretation of the measurements. Highly contoured surfaces reflect the light back to the device in different angles, which might create artifacts and make interpretation of the images impossible [44]. A change in the ambient temperature range might affect the measurements as well. Previous and/or current smoke exposure was related to higher basal StO_2_ in the assessment of peripheral microcirculation in scleroderma [45]. Therefore, changes in tissue oxygen saturation should be evaluated during the distraction period rather than the absolute values.

Furthermore, we present just a small number of patients who were treated with this device. Individual characteristics, for example the stadium of the disease or if it is a recurrent disease, have a higher impact on the evaluation in such a small group. However, since not many patients present with far-advanced flexion deformities of a finger joint that needs slow distraction, a much higher number of treated patients will be difficult to achieve. Nevertheless, a larger prospective study would be helpful to elucidate the positive effect of this device.

To the best of our knowledge, this is the largest study that evaluates the use of NIRS in the context of skeletal distraction of contracted joints in DD.

## 5. Conclusions

The application of NIRS can enhance safety in the treatment of far-advanced DD with an external skeletal distraction device. It is non-invasive, easy to use and does not require specific training.

## Data Availability

Due to the nature of this research, participants of this study did not agree for their data to be shared publicly, so supporting data is not available.
